# Reconnected project in Kosovo: first steps in co-creation process

**DOI:** 10.1192/j.eurpsy.2025.1424

**Published:** 2025-08-26

**Authors:** N. Fanaj, S. Mustafa, E. Krasniqi, A. Cerga Pashoja

**Affiliations:** 1 Alma Mater Europae Campus College Rezonanca, Prishtina; 2 PMSH, Prizren; 3 AAB College; 4 UBT College, Prishtina, Kosovo; 5 Faculty of Sport, Technology and Health Sciences, St Mary’s University, London, United Kingdom

## Abstract

**Introduction:**

Current global societal challenges pose significant threats to the mental well-being of European citizens. The ‘RECONNECTED’ project, supported by the EU/Horizon Europe program, aims to tackle mental health issues experienced by vulnerable populations in Europe, including those in Kosovo, particularly individuals with low socioeconomic status.

**Objectives:**

The objective is to present key pillars of project and outline some key findings that will inform the consortium toward finalizing the conceptual framework of RECONNECTED which draws inspiration from the Urban Mental Health (UMH) framework, with a focus on particular aspects related to Kosovo.

**Methods:**

It’s a mixed-methods study design. We have reviewed the literature related to three key pillars of project: social prescribing, mental health literacy and personalized micro-interventions in Kosovo, and analysed preliminary some recent cross-sectional samples focusing on sociodemographic and psychosocial variables. Data processing was done with SPSS 27.0 and Microsoft Excel 2019.

**Results:**

The literature review in internet does not find information about the implementation of social prescription and personalized micro-interventions in Kosovo so far. However, we can see that similar fragmented activities of social prescription have been implemented in Kosovo in various unstructured and informal designs. There is also information about the individual use of various applications by young people. Furthermore, no study is found regarding mental health literacy in Kosovo, and we have found that there are no mental health promotion programs. Recent cross-sectional samples analysis highlights the association between gender, dysfunctional coping style, social support and low socio-economic status with mental health indicators like anxiety, depression and suicidal ideation among young individuals.

**Conclusions:**

These findings highlight how key mental health challenges could impact the implementation and use of a digital support system like RECONNECTED within a community care model. Addressing these factors during the co-creation process between researchers, stakeholders and end-users is crucial for ensuring the success of this innovative scientific approach in Kosovo, a low-middle-income country.

**Disclosure of Interest:**

N. Fanaj Grant / Research support from: Principal Researcher for Kosovo in Project RECONNECTED, S. Mustafa Grant / Research support from: Researcher Officer for Kosovo in Project RECONNECTED, E. Krasniqi Grant / Research support from: Researcher Officer for Kosovo in Project RECONNECTED, A. Cerga Pashoja Grant / Research support from: Principal Researcher for UK in Project RECONNECTED

